# Lifelong Fitness in Ambulatory Children and Adolescents with Cerebral Palsy II: Influencing the Trajectory

**DOI:** 10.3390/bs13060504

**Published:** 2023-06-15

**Authors:** Susan V. Duff, Justine D. Kimbel, Marybeth Grant-Beuttler, Theresa Sukal-Moulton, Noelle G. Moreau, Kathleen M. Friel

**Affiliations:** 1 Department of Physical Therapy, Crean College of Health and Behavioral Sciences, Chapman University, Irvine, CA 92618, USA; jkimbel@chapman.edu; 2 Department of Physical Therapy, Oregon Institute of Technology, Klamath Falls, OR 97601, USA; marybeth.grantbeuttler@oit.edu; 3 Department of Physical Therapy & Human Movement Sciences, Northwestern University, Chicago, IL 60611, USA; theresa-moulton@northwestern.edu; 4 Department of Physical Therapy, Louisiana State University Health Sciences Center, New Orleans, LA 70112, USA; nmorea@lsuhsc.edu; 5 Burke Neurological Institute, Weill Cornell Medicine, White Plains, NY 10605, USA; kaf3001@med.cornell.edu

**Keywords:** cerebral palsy, motor learning, lifestyle intervention, lifelong fitness

## Abstract

Physical activity of at least moderate intensity in all children contributes to higher levels of physical and psychological health. While essential, children with cerebral palsy (CP) often lack the physical capacity, resources, and knowledge to engage in physical activity at a sufficient intensity to optimize health and well-being. Low levels of physical activity place them at risk for declining fitness and health, contributing to a sedentary lifestyle. From this perspective, we describe a framework to foster a lifelong trajectory of fitness in ambulatory children with CP (GMFCS I–III) as they progress into adolescence and adulthood, implemented in conjunction with a training program to augment bone and muscle health. First, we recommend that altering the fitness trajectory of children with CP will require the use of methods to drive behavioral change prior to adolescence. Second, to promote behavior change, we suggest embedding lifestyle intervention into fitness programming while including meaningful activities and peer socialization to foster self-directed habit formation. If the inclusion of lifestyle intervention to drive behavior change is embedded into fitness programs and found to be effective, it may guide the delivery of targeted programming and community implementation. Participation in comprehensive programming could alter the long-term trajectory of musculoskeletal health while fostering strong self-efficacy in persons with CP.

## 1. Introduction

Exercise and fitness are key elements of lifelong physical and psychological well-being and have been referred to as “the fountain of youth” [1]. Multiple public health and professional organizations recommend that children and adolescents from 6 to 17 years of age engage in moderate to vigorous exercise for 60 min per day at least 3 times per week, including activities for bone and muscle strengthening and aerobic conditioning [2,3,4]. Physical activity in this context refers to the bodily movements performed by skeletal muscles that result in energy expenditure [5]. Exercise can be considered a subset of physical activity, particularly when the aim is physical fitness and the activity is structured, planned, repetitive, and goal-directed.

The amount and intensity of physical activity strongly influence physical and psychosocial health in typical and atypically developing children. Daily physical activity has been found to improve a child’s self-esteem, social interactions, and stress management [6]. In a study of over 4000 typically developing (TD) children 8–10 years of age, Vella and colleagues [7,8] found that ongoing participation in organized extracurricular sports was associated with higher levels of social and emotional health by age 10. Conversely, children who are inactive may be at higher risk for chronic health conditions such as cardiovascular disease and diabetes, which negatively influence physical and psychological well-being as they age [9]. These risks are even higher for those with physical disabilities such as cerebral palsy (CP), with the effects of inactivity compounding as they progress through adolescence into adulthood [10,11,12].

The purpose of this perspective paper is to describe a framework of lifestyle intervention designed to foster autonomy, motivation, social engagement, and personal habits to run in conjunction with a program aimed at promoting bone and muscle health in children with CP at GMFCS levels I–III [13]. To aid the identification of key ingredients essential to successful fitness programming for children, we surveyed youth with and without CP who participate in physical activity regularly and one parent each to gain their perspectives. The themes generated from the surveys are reviewed below. With sufficient evidence regarding methods to safely augment bone and muscle health among children at GMFCS levels IV–V and how to best engage children of varying intellectual capacities, we hope to expand our program to include a wider range of children with CP. It is our view that if children with CP at any level can feasibly establish an active, self-directed, fit lifestyle by adolescence, it could foster the development of healthy personal habits that may extend to adulthood.

## 2. Physical Activity and Fitness

In general, children with CP have lower levels of physical activity than TD children [6,14]. Individuals at levels IV–V, based on the Gross Motor Function Classification System (GMFCS), have much lower levels of physical activity than those at GMFCS levels I–III who can ambulate with or without an assistive device [15]. Based on data gathered from 3 days of wearing wrist and ankle accelerometers and self-reported daily logs, children with CP at GMFCS levels II–III were found to have lower levels of daily active energy expenditure in comparison to children who were TD [6]. In another study [14], walking activity was tracked in persons 10–13 years of age with CP at levels GMFCS levels I–II and youth who were developing typically. Data were gathered from all participants for 7 days while they wore a StepWatch monitor (Mountlake Terrace, WA, USA) unilaterally on one ankle. The authors found that the youth with CP were significantly less active than their TD peers [14]. Yoon and colleagues [15] assessed physical activity in children with CP, aged 4–18 years of age, across GMFCS levels I–V with varying comorbidities. The participants wore a GT3X+ (ActiGraph, Pensacola, FL, USA) accelerometer on the waist for 7 days. The findings revealed that non-ambulatory children at GMFCS levels IV–V spent significantly less time in moderate to vigorous activity than ambulatory children at GMFCS levels I–III. Decreased physical activity was also associated with lower physical quality of life (QOL). These data represent an overall trend of decreased activity that contributes to secondary health conditions [16,17], even as the lifespan of persons with CP continues to expand [18]. Therefore, to best promote a healthy lifestyle that could minimize the development of chronic conditions we need to better understand the pace at which motor function decreases [17] and how to successfully engage children in fitness at a young age, to promote long-term adherence to physical activity.

There are many challenges that children with CP may face when striving to reach sufficient physical activity and fitness. These concerns must be addressed in programming to ensure adherence and engagement. First, given their physical disability, they are often less active and more reliant on external resources to facilitate participation in sports and other activities, making self-advocacy more onerous while placing an additional burden on parents. Second, bias from others and a lack of program accommodations can limit their inclusion in extracurricular sports programs, even for those at higher functioning GMFCS levels compared to age-matched peers without physical disabilities. Finally, without clear personal choice and access, children with CP may feel disconnected and isolated from their community [19], leading to underdeveloped social skills and insufficient resilience to stress. As these children move into adolescence, these effects may contribute to lower levels of self-efficacy [6,20], defined as an individual’s confidence that they will be able to perform actions that bring about desired task outcomes [21]. These and other barriers often limit the establishment of healthy habits essential to the development of an active, fit lifestyle [8,19]. However, if these barriers were minimized and meaningful skills were developed, self-efficacy may improve. While overcoming challenges and promoting fitness habits to minimize the adoption of a sedentary lifestyle are key, it is important to consider how programs that aim to augment bone and muscle health with lifestyle intervention embedded in them can be successfully implemented.

## 3. Altering the Fitness Trajectory of Children with CP

Investigating the factors that affect an individual’s willingness to participate and adopt positive fitness habits is vital to long-term adherence. González-Hernández and colleagues [22] showed that vigorous consistent exercise and the pursuit of perfectionism in teenagers who engaged in sport-centered or recreational exercise resulted in increased self-efficacy and psychological well-being. This so-called adaptive perfectionism may encourage adherence to regular fitness activity due at least in part to the positive outcomes that result. In a longitudinal study of 8–10-year-old TD children, Vella et al. [7,8] found that organized, consistent, and developmentally appropriate sports and physical activity were linked with higher levels of health-related QOL. Positive outcomes that result from successful participation often encourage frequent engagement in regular fitness activity and higher overall levels of well-being.

There is no physiological reason to anticipate that these same results would not be possible in children with CP who are able to increase their level of physical activity. Fortunately, the availability of participation-based community exercise programs for persons with CP is increasing, particularly for those at GMFCS levels I–III, and positive outcomes are being reported [20,23,24,25,26]. Cleary and colleagues [23] conducted an aerobic training study for youth with CP in a school setting, showing evidence of high adherence and significant gains in cardiovascular markers with no adverse events for the exercise group. Thorpe et al. [25], evaluated the outcomes of a treadmill training or aquatic exercise program held at a community center for adolescents with CP at GMFCS levels I–III. The authors found increases in walking distance, leg muscle mass, and self-perception of function among all participants. A 6-week ballet program was run for youth with CP, aged 9–14 years, at GMFCS levels I–III [26]. The results revealed improvements in select gait parameters including decreased time of ambulation and increases in step and stride length [26]. The evidence for successful outcomes from community programs for children with CP continues to expand but adherence and the prevention of a sedentary lifestyle remain challenges that must be addressed.

**Figure 1:**
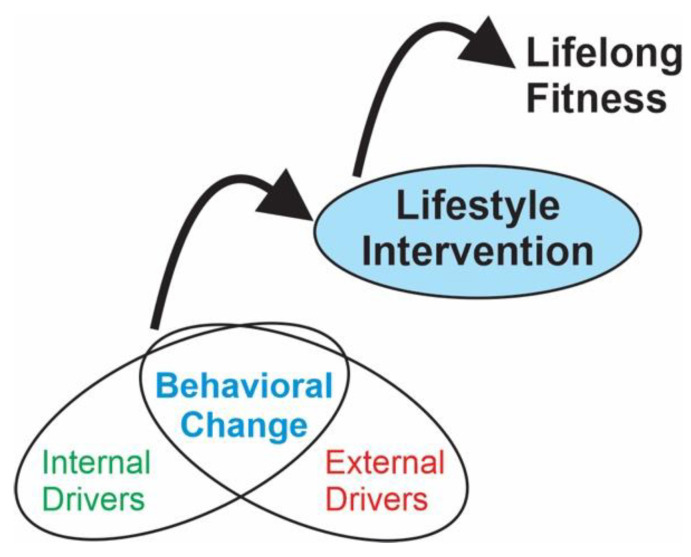
Framework for changing behavior via lifestyle intervention to achieve lifelong fitness. Internal drivers—system integrity, self-determination, and skill acquisition. External drivers—opportunity and relationships. With lifestyle interventions embedded into meaningful program activity and socialization, self-initiated habits could contribute to lifelong fitness.

The development of the key ingredients for successful long-term participation in fitness activities should begin in childhood. In addition to the inclusion of methods to enhance bone and muscle health, programs could incorporate components to drive behavioral change, fostered through lifestyle intervention (see Figure 1). With strong evidence and clinical expertise, practical program guidelines can be designed to improve specific outcomes while assessing for effectiveness and adherence in children with CP across all GMFCS levels of classification [27,28].

### 3.1. Internal Drivers of Behavioral Change

Despite the challenges, most persons with CP do wish to be active, fit, and socially connected [29]. Altering the fitness trajectory of children with CP may require attention to the internal or individual drivers of behavioral change. Studies stemming from select psychological theories and motor learning principles can serve to guide this aspect of program design to bolster long-term adherence to physical activity and fitness.

Self-determination theory: This theory proposes that intrinsic motivation is fostered when three basic psychological needs are met: autonomy, competence, and relatedness [30,31]. According to self-determination theory (SDT), *autonomy* refers to a sense of initiative and choice; *competence* signifies a feeling of mastery or success; *relatedness* denotes a sense of belonging or connection [31]. Autonomous forms of motivation can positively predict the likelihood and duration of exercise participation [32]. Feelings of competence stemming from experiences of mastery clearly influence overall well-being and the ability to sustain behavior change. Relatedness acquired through accepting environments and meaningful personal relationships can strongly motivate participation. Conversely, external control, harsh internal or external critiques of performance, and an inability to relate to other participants or coaches can limit behavior change and negatively influence participation. Therefore, to foster motivation using SDT, participants should make activity choices and task demands that are achievable with minimal negative feedback within programs that are positive and accepting. Fostering a child’s social connections while promoting skill development and active learning can strengthen engagement in fitness programs, which promote behavior change [19]. Since motivation is an important aspect of behavioral change, incorporating aspects of self-determination theory within programming may be essential [33].

Skill acquisition: Skills are actions that demonstrate consistency, efficiency, and flexibility [34]. *Consistency* refers to the repeatability of a task or skill over time. *Efficiency* refers to the optimization of energy resources from the musculoskeletal and cardiovascular systems. *Flexibility* or transferability refers to the adaptability of task or skill performance to changing environments or conditions. Specific aspects of skill acquisition could be incorporated into training programs to foster motor learning. Considerations include understanding the stage of the learner, structuring the task practice, and the strength of intrinsic and extrinsic feedback, among others.

When designing programs to enhance skill acquisition, one should first consider whether the individual is in the early or later stage of learning a particular sport or activity [35,36]. In the early stage of learning, a child would be developing an understanding of the task goal and the dynamics (i.e., the power required to throw a ball to a target). They would be developing movement strategies and learning to distinguish between the regulatory and non-regulatory features of the environment. Regulatory features are conditions to which the movement must conform, such as the size and speed of a moving baseball when catching. The non-regulatory features are those that can influence skill performance but the body does not have to conform to them, such as the color of the baseball. In the later stage of learning, a child would be refining movements, adapting to changing tasks and environmental demands. They would be learning to perform tasks consistently and efficiently. Improving a physical skill in a context that allows an individual to modify the force used or spatial–temporal requirements to perform the task or skill enhances flexibility or transferability.

Setting up the practice conditions and ensuring that the feedback is sufficient during task performance are elements that can be tailored to any stage of learning [37,38]. The practice conditions for a particular skill or task can differ in their amount, the order of specific components, or whether part or whole practice will be used. The practice could be specific to one task and done in ways that vary the amount a child receives. Practice can be done in one intensive (massed) bout, such as a boot camp type experience, or it could be distributed across time, such as soccer practice for one hour per session, two sessions per week, for many weeks. Practice can be scheduled in a random or blocked order within a day or over a set period of time. Children could practice a full activity, such as playing games of baseball, or could repetitively practice one aspect of the activity, such as throwing a baseball repeatedly. Part-practice should ideally be followed by practice of the whole activity. Preferably, the practice conditions should be individualized.

Feedback can be intrinsic or extrinsic, providing knowledge of performance or knowledge of results. Intrinsic feedback is the sensory experience gained through the movement itself. Extrinsic or augmented feedback can be verbal, visual as with demonstrations, or physical as provided with manual guidance. Biofeedback is an additional form of augmented feedback. Extrinsic feedback can be given concurrently during practice or at the end of performance. It can be provided 100% of the time or less. It may be precise or general. The research suggests that extrinsic feedback should be given intermittently and should lessen over time [39,40]. We propose that to foster physical and behavioral changes that promote long-term adherence to fitness, motor learning principles should be incorporated into programming and measured by retention or transfer tests [34].

The Optimal Theory: This theory introduced by Wulf and Lewthwaite [21] proposes that performance and motor learning can be optimized through intrinsic motivation and attention, linking goals to actions. The three key factors of this theory are *enhancing expectancies* for future performance, *autonomy*, and *an external focus* of attention on motor actions. Theoretically, performance can be enhanced by including statements such as “children who exercise every day often get stronger,” encouraging the adoption of consistent physical activity. Autonomy refers to the choices one has, such as allowing the child to choose the sequence of program activities. An external focus of attention refers to attention on a target versus a focus on bodily actions. The authors [21] propose that dopamine responses increase due to the anticipation of positive experiences, which could contribute to motor learning [41,42]. Integrating this theory into programming can help foster motivation, autonomy, and attention.

Instilling self-determination and autonomy is vital to developing and maintaining fitness habits in children with CP as they move through adolescence into adulthood [32]. Palisano et al. [19] noted that the autonomous control of decisions, flexible and individualized approaches, and opportunities for problem-solving at an incremental level are key factors in a self-determined strengths-based approach to fitness participation. The activities should be intentionally designed to promote autonomy while providing the necessary structure and support for successful task completion. A segment of any program could be individualized and self-directed to raise a child’s self-efficacy and satisfaction in their own competence. For example, if a child wishes to play soccer, the part-practice of essential aspects of the game followed by whole practice could be integrated into their training program. According to the optimal theory, providing choices aids motivation. Thus, offering a choice regarding the part-practice order, such as between high-velocity power training of the legs, ball passing strategies, or shooting goals, may provide an incentive. Then, after the part-practice of all 3 aspects of soccer, a one-on-one game could be played with a peer or sibling. This type of sport-specific training within a fitness program could encourage the adoption of essential physical skills and learning in a natural environment. Ensuring self-determination and personal choice could aid investment in future goal setting and contribute to a positive fitness trajectory [31,32,33].

### 3.2. External Drivers of Behavioral Change

Factors external to the individual often have a strong influence on behavior change. Initially, there must be the opportunity to engage. Then, altering the fitness trajectory of children with CP would require a focus on external drivers of change, such as the task and environment [38] and relationships with parents, peers, and mentors. These external factors must be considered in combination with internal or individual factors.

#### 3.2.1. Opportunity

Challenges or adversity in tasks and the environment can contribute to the development of psychological resilience. Within the environment, one must consider whether it is open or closed. In a closed environment, the environment is stable, as when walking up a standard set of stairs. An open environment has time constraints and involves prediction, such as for the speed and location of moving objects when trying to step up onto an escalator or catch a moving ball. When planning tasks, providing “just the right challenge” [43] is a common phrase used to foster success in training programs for children. Along with this, it is important to consider affordances, defined by Gibson [44] as the reciprocal fit between the person and the environment needed to perform tasks. For example, when learning to catch balls, trainers may start with large balls or large mitts so that the allowable bandwidth of error is wider. Practicing new skills that incorporate participant strengths may reframe challenging tasks and activities from obstacles to opportunities for growth [19].

#### 3.2.2. Relationships

Parents: Parents are a crucial domain of influence in a child’s successful participation in physical activities and fitness programs. In a qualitative study interviewing parents of 8–11-year-old children with CP, Lauruschkus et al. [45] reported that parents desire opportunities for their child to have peers with whom they can be physically active. Additionally, they found that parents tend to seek programs where competent persons can provide support for participation. Family culture and attitudes towards fitness, the level of support in facilitating participation, and the feasibility of the program (location and frequency) are important factors to consider in designing a fitness intervention program.

Peers: Sport-based youth development is a program strategy that aims to promote healthy behaviors concurrently with social confidence [46]. This is often achieved through team building and athletic games while increasing resilience and the ability to handle adversity, which are important components for developing social–emotional well-being in children. Peers provide a motivating avenue to participation, adding *“fun”* to activities given the natural flow between children, perhaps because they identify with each other due to having similar interests and communication styles. Encouraging participation in fitness programs in pairs, whether with siblings or age-matched peers, could enhance engagement and foster a sense of belonging, promoting social confidence.

Mentors: The acknowledgement of progress checkpoints toward a larger goal may increase self-driven participation and encourage adherence to that goal, especially in the context of skill-learning and physical ability. Mentors and coaches can also be models for certain tasks, demonstrating feasibility while providing encouragement. Effective interventions involve collaborative goal-setting among the child, family, and coach. Helping children distinguish between where they are now and where they want to be could enhance their motivation to achieve short-term goals as precursors to larger goals [47].

## 4. Engaged Consumers

While the literature provides a theoretical underpinning of how to promote a positive trajectory, there are practical implications where additional feedback is needed. To better inform the direction of a new program, we surveyed 11 youth, 8 to 18 years of age with and without CP, along with one parent or guardian each to determine their perspectives regarding the most desirable rewards and the feasibility of conducting a fitness program at a sufficient frequency to improve bone and muscle health. We hypothesized that the reward preferences and feasibility would differ between children of different ages, whether the person is TD or has CP, and where they reside. All youth participants were recruited because of their successful participation in physical activities. The children with CP were within GMFCS levels I–II. The youths and parents both completed an online questionnaire (Appendix A) pertaining to internal and external factors of influence to better understand features of motivation and reward as well as obstacles to participation [48].

Participant (youth) results: The survey indicated that 75% of youth participants reported a preference for activities that were not physically active, including computer games or hanging out with friends. However, 62.5% ranked physical activity as “essential” or “very important” for their health. All participants (100%) believed that time spent exercising would result in positive physical changes. Over 62% reported that activity with a goal to improve strength would motivate them to participate in a fitness program, whereas 50% were motivated to participate in programs that improved endurance or coordination. The survey indicated that most children (75%) felt that healthy bones and muscles required activity on a “frequent” basis and every participant indicated they would complete activities that were not fun if it helped them to improve.

Fifty percent of participants reported that physical activities involving friends were a positive motivator to participate. However, twenty-five percent indicated that situations that highlighted their limitations in “keeping up” with others negatively influenced their desire to participate. Additional limiting factors to participating in physical activity included transportation, with most children reporting they would be reliant on a parent to transport them to and from any activity outside of school. Time was also reported as a limiting factor, stating that school and homework were their highest priorities.

Parent results: Nearly 66% of parents were interested in physical activity programs that focused on improving their child’s strength, agility, and flexibility. However, this same number of parents reported that they were not aware of the current programming recommendations for bone and muscle health. Fifty percent of parents reported that they had concerns about their child participating in physical activity programs. When asked to elaborate on their concerns, parents reported that they were concerned about the time required to participate in a program, transportation to get their child to the program, and safety for their child’s “specific needs”. The large majority of parents (87.5%) would be willing to drive their child to a fitness program, with 50% willing to drive to programs run 3 times per week for 3 months or longer.

**Figure 2:**
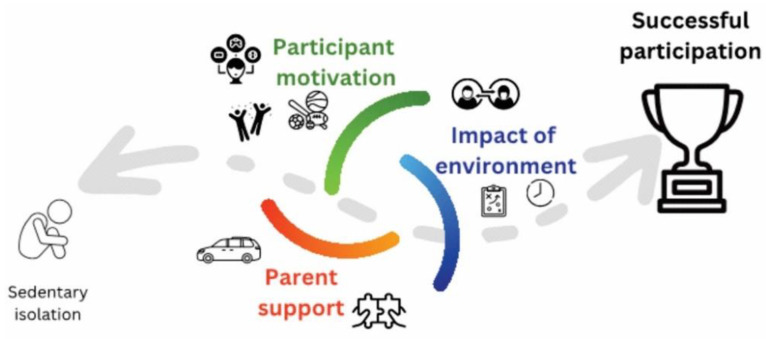
Domains of influence toward successful community participation.

Our analysis of participant and parent survey responses supported the three main domains of influence regarding the successful participation in physical activity and fitness for these interviewees: the participant, parent, and environment. Based on the literature and our findings from the surveys, we developed a simple yet clear model featuring key domains of influence to participation in children and adolescents with CP (see Figure 2). These domains of influence for participation were given strong consideration in the design of our framework to foster lifelong fitness.

Participant domain: Most of the children who represented successful examples of participation clearly revealed *competence* in their survey responses based on the self-determination theory [31,32]. The responses indicated that they desired to improve their physical skills and that this improvement was important to them, recognizing its positive contribution to long-term performance goals. The participants reported negative feelings of competence when they felt that their physical performance was judged as poorer than their peers. This suggests that self-perception of competence in select activities can be uplifting or manifest as an aversion to failure if a participant believes they will be judged unfairly by others.

Parent domain: Many parents are interested in programs and activities that they perceive as focused on areas of weakness for their children. However, it appears that there is a need to educate parents on current recommendations on bone and muscle health and the need to foster an internal drive in participants. Negative factors for parents include programs that do not “match” their child’s physical abilities and require significant time and travel. They often reported feeling hesitant and responsible for providing accessibility for their child. The barrier to a parent’s investment in a child’s activities [49,50] can be addressed by a supportive community with consideration for the timing of sessions, carpooling options, and convenient facility locations. By supporting the parents and aiding them in overcoming barriers, the child can receive support for their autonomy in participation [49,51].

Environmental domain: The impact of the environment was the third domain of influence brought up by participants. Factors of program frequency and location were the most common influences on activity participation. All youth expressed that they would prefer to participate in outdoor spaces or gyms and would be willing to commit to a program for about three months. The parents expressed that the location of the program matters but most would be willing to support their child’s participation even if long commutes are required. The main concerns about participating in a fitness program raised by both youth participants and parents were scheduling conflicts and the level of difficulty. We believe that transitioning the motor skills practiced and learned in a simulated environment to real-world tasks could lead to higher levels of community participation among children with CP.

## 5. Lifestyle Intervention

Without personal investment in altering one’s lifestyle, any changes in activity level and fitness may be short-lived. Lifestyle Redesign^®^ is a therapeutic means of enabling people to actively engage in individualized health-promoting occupations [52,53,54,55] while limiting their reliance on external factors. Specifically, the design of an intervention to drive behavior change linked to exercise and physical activity must satisfy the lifestyle needs of the participants and can include education and coaching on themes that are meaningful and important to the participants.

Lifestyle intervention has been successfully used to design programs for improving physical fitness in children with CP [56,57]. In a randomized controlled trial by Slaman et al. [56], adolescents at GMFCS levels I to IV engaged in 3 months of fitness training and 6 months of counseling on daily physical activity and sports participation. The authors found improvements in cardiopulmonary fitness, muscle strength, and body composition after the physical fitness intervention. However, the short-term success in adherence to physical fitness was no longer seen six months after the intervention. Despite the early gains, it is important to consider the missing ingredients in programming. The suggestions for future programming from the authors were to include accelerometry to provide immediate feedback to participants. They also revealed that if parents considered involvement in fitness programming more important than their children did, this led to drop-out. Based on the findings from this study, it seems essential to foster self-determination and personal fitness habits within a lifestyle intervention program to achieve sufficient adherence to training and move toward a positive, long-term fitness trajectory.

Determining which activities are meaningful to children as well as their perceptions of their ability should be assessed from the onset of any fitness training program. Therefore, prior to engaging in any training program, we recommend having children rate their performance and satisfaction on meaningful and desirable skills and fitness using a tool such as the Canadian Occupational Performance Measure (COPM) [58,59]. To measure changes in perceptions of self-competency and self-efficacy among participating individuals, we also recommend the Children’s Self-Perceptions of Adequacy in and Predilection for Physical Activity (CSAPPA) [60,61]. A coaching tool such as motivational interviewing (https://motivationalinterviewing.org/) [62,63], which involves active listening, open questions, and affirmations of strengths and past successes, may encourage the child to make decisions based on their own reflections during or after training. Using an approach titled solutions-focused coaching [64,65,66], Schwellnus et al. [64] found improvements in goal satisfaction, attainment, and performance based on the attainment of participation goals in a group of 12 children with CP at 6–19 years of age. With this relatively small sample size, the authors used quantitative data, the COPM, and the Goal Attainment Scale to evaluate progress on short-term goals. Further research is needed to investigate the effects of the solutions-focused approach on long-term goals, yet the findings suggest that it may be effective in improving participation goals among children with CP [64]. Programs that include personal goals with guidance from program leaders and those that embrace challenges as a link to goal achievement while fostering resilience in facing obstacles should be included to ensure success. Including activities that are meaningful and achievable can aid in the development of key skills inherent in activities or sports of individual interest in any fitness program.

Based on the evidence and results from our survey, we are designing a fitness program that aims to target bone and muscle health through the optimal dosing of training exercises [13,67,68] for children at GMFCS levels I–III, with consideration of internal and external drivers of behavior change fostered through lifestyle intervention. The combination of these important aspects of programming will ideally foster self-directed habit formation with the overarching goal of promoting a positive, lifelong fitness trajectory in these children with CP (see Figure 1). The evidence base is primarily available for the use of these techniques in children who are able to actively participate in higher gross motor skill types of physical activity (GMFCS I–III). However, given the heterogeneity of CP, investigations with a more inclusive range of participants and with respect for their life experience is necessary. With greater evidence and safe guidelines, future research studies and programs such as ours could be more inclusive, with a focus on this underrepresented population.

## 6. Lifelong Sustainability

Introducing sustainable physical activity options at an early age that could augment musculoskeletal health is essential for persons with CP. To ensure that fitness is maintained into adolescence and adulthood, such programs must also be engaging and fit into one’s interests and lifestyle. We propose that a comprehensive, individualized program introduced in pre-adolescence will provide the optimal stimulus to enhance the integrity of multiple systems, prevent the acquisition of a sedentary lifestyle, and contribute to positive self-efficacy. Ideally, programming should be integrated into one’s lifestyle and have a positive link to function and skill acquisition. Importantly, building accepting, integrated fitness communities is essential for people with CP and other disabilities. If a person is active in childhood and adolescent fitness programs tailored exclusively to people with disabilities, they may easily become frustrated when they age out of adaptive programs and lack the confidence and tools to interface with non-adaptive programs. As acceptance by the non-disabled community and physical access continue to be quite variable, it is important to teach self-advocacy to young people with disabilities.

Despite the risks of developing a sedentary lifestyle, if the physical activity required to significantly enhance and maintain musculoskeletal health could be incorporated at an earlier age, this trend may be altered. This could begin by having a child or adolescent make the choice regarding which physical activity or sport they wish to be involved in and committing to opportunities to engage in that activity. Choice alone may help to increase the level of engagement and degree of skill acquisition [69]. Since most individuals thrive on socialization and companionship, programs that include these aspects are likely to be more readily accepted. If a personal goal to engage in community programs is known, the ingredients of an exercise program can include the essential motor skills needed to achieve the goal.

While exercise programs can improve motor function, they can also increase the readiness for participation in community-based activities if methods to foster internal motivation are embedded into programming. As cited earlier, Thorpe et al. [25] conducted a treadmill training and aquatic exercise program at a community center for adolescents with CP. The authors found that along with improvements in outcomes, having the program at a community center was beneficial for both the adolescents and the staff/members of the center. The authors believe more research is needed on the role motivation plays in lifelong physical activity for individuals with CP [25]. Another study by Darrah et al. [70] found that a fitness program held at a community center increased muscle strength and perceived confidence in adolescents with CP. By organizing a fitness program into pairs or peer groups, socialization and teamwork become essential and can lift engagement and the readiness to participate in other community activities.

### Case Example

A co-author of this manuscript is an adult with CP (K.F.). In her mid-30s, she noticed that her balance, strength, and endurance were decreasing compared to when she was younger. She realized that she needed to increase her activity level to maintain her health. K.F. decided to begin martial arts training and joined the Harlem Tae Kwon Do (TKD) family as its first student with CP. Each class began with stretching and strength-building exercises that are similar to activities she did, unexcitedly, for years as a child and adolescent in physical therapy. By engaging in the same principles of building flexibility, strength, coordination, and balance at a TKD program, she greatly improved her fitness level beyond what she felt as a young adult. In addition, TKD classes are fun, challenging, and build community, and K.F. quickly made friends in the program who offer an added layer of engagement and accountability—if she misses too many classes, she is contacted and encouraged to return! These key ingredients of fun, accountability, community, and a variety of engaging exercises have given K.F. greater flexibility, balance, strength, and confidence in her body. This case shows how it is possible for people with disabilities to harness the ingredients for life-long fitness. By identifying and incorporating these ingredients into programming, K.F.’s successful participation could be replicated by others. As shown, exercise that is valuable and enjoyable could be the ‘bridge’ to sustained fitness for its physical and psychological benefits.

## 7. Conclusions

Our framework shown in Figure 1 aims to incorporate the ingredients for lifelong fitness and directly fulfill the psychological needs for autonomy, relatedness, and competence in order to increase self-efficacy and internal motivation. Framing a fitness program as a gateway to a fit and active lifestyle rather than a means to an end, such as receiving a tangible reward, could enhance adherence to fitness. Thus, recognizing opportunities for both physical and psychological growth and improvement are fundamental to fostering a lifestyle of fitness and healthy habits, which could be achieved using a framework of lifestyle intervention.

Improvements in bone and muscle health, motor skills, exercise habits, and the development of internally driven motivation may strongly influence the long-term adherence to fitness in children with CP. Including aspects of our suggested domains of influence toward motivation and participation could increase the adoption of positive fitness habits among children with CP. If children have not been fully engaged in physical activity, they need to find a sport or activity that is desired or gives them the most satisfaction on a social and physical level. While this would ideally be attained in pre-adolescence, it is not always done. An improvement in self-efficacy can provide reference and structure for an active lifestyle that may not have been recognized prior to participation in a targeted fitness program. If found to be feasible and effective, our framework for lifestyle intervention embedded within programs designed to augment bone and muscle health could be implemented into the community before adolescence so that we could truly alter the fitness trajectory of children with CP.

## Data Availability

Requests for survey data can be made to duff@chapman.edu.

## Appendix A. Fitness Program Incentive Questions—Program Ideas

Start of Block: Part 1: Child

Child name (first):____________________________ Age: _____________

1. What is your favorite thing to do outside of school?

________________________________________________________________

________________________________________________________________

________________________________________________________________

2. Do you to do things that make you move around and/or breath hard? (Select One)

_____ Yes (1)

_____ No (2)

3. What physical activity (sport, dance, martial art, playing outside) do you participate in most outside of school? Why do you like it?

________________________________________________________________

________________________________________________________________

________________________________________________________________

4. Where do you participate in this activity or exercise? At home, school, or somewhere else?

________________________________________________________________

________________________________________________________________

________________________________________________________________

5. If you participate outside your home or school, how do you get there?

________________________________________________________________

________________________________________________________________

________________________________________________________________

6. Was it your idea to start doing this activity or exercise? (Select one)

_____ Yes (1)

_____ No (2)

*Display: Was it your idea to start doing this activity or exercise? (Select one) = Yes or No*

7. If not, who suggested it?

________________________________________________________________

8. What do you need to get better at this activity or exercise? Select all that apply.

____ Move faster

____ Be stronger

____ Keep moving for longer

____ Be more flexible

____ Be more coordinated

____ Other: __________________________________________________

9. Do you believe working out will help you get better now or in the future? (Select one)

____ Yes (1)

____ No (2)

10. Rank the following physical activities in terms of your interests. Drag to rank each item in order 1 (best)–5 (worst):

____ Playing outside

____ Playing sports

____ Strength exercises (push-ups, sit-ups, etc.)

____ Dancing

____ Martial Arts

11. How important is it to you to be fit or have an active lifestyle (pick one)?

____ Essential

____ Very important

____ Important

____ Slightly important

____ Not at all important

12. What would you be most excited about if you were able to do a fitness program?

________________________________________________________________

________________________________________________________________

________________________________________________________________

13. Does anything about a fitness program worry you?

________________________________________________________________

________________________________________________________________

________________________________________________________________

14. Do you think having the right type of gear (clothes, water bottles, sneakers, etc.) is important to participate in a fitness program?

________________________________________________________________

15. Are you willing to do activities that are less fun if they help you get better at sports or activities you like to participate in? (Select one)

____ Yes (1)

____ No (2)

16. Rank order the list of rewards below by what you would prefer, Rank in order of 1 (best)–7 (worst):

____ Free game apps for my phone or computer

____ Favorite food treats like

____ Free tickets to a movie, play, or concert

____ Free entry into an amusement park

____ Free months at the gym, dance program, martial arts program or other site

____ Free gear for participating in activity of choice

____ Other rewards: ________________________________________________

17. What would motivate you to get more fit? Rank in order of 1 (best motivator)–6 (worst):

____ Having a friend or brother/sister do it with me

____ Go to a fitness center or site that other kids go to

____ Get better at my sport, dance, martial art, or other activity

____ Trying new activities

____ Receive rewards

____ Other

18. How often do you think children should exercise to improve their bone and muscle health?

(Select one)

____ Frequently

____ Sometimes

____ Not frequently

19. How many times per week would you be willing or able to go somewhere outside your home to improve your fitness? Consider other things you do, such as homework or music lessons.

____ 1/week

____ 2/week

____ 3/week

____ More

20. Would you be willing to participate in a fitness program for 3 months or more? (Select one)

____ Yes (1)

____ No (2)

21. What would be the biggest challenge for you to participate for 3 months or more?

________________________________________________________________

________________________________________________________________

________________________________________________________________

22. Other comments:

________________________________________________________________

________________________________________________________________

**End of Block: Part 1: Child**
**Start of Block: Part 2: Caretaker**
Caretaker Name (first): ________________________

23. What physical activities or fitness programs would you like your child to be involved in?

________________________________________________________________

________________________________________________________________

________________________________________________________________

24. What is your view of your child’s current physical activity or exercise program? How do you support your child’s participation in physical activities?

________________________________________________________________

________________________________________________________________

________________________________________________________________

25. Does anything about a fitness program that involves or would involve your child concern you? (Select one)

____ Yes (1)

____ No (2)

Display: *Does anything about a fitness program that involves or would involve your child concern, you? = Yes or No*

26. If you answered yes to the question above, what would the concern(s) be?

________________________________________________________________

________________________________________________________________

________________________________________________________________

27. What would motivate your child to get more fit (rank these)? 1 (best)–6 (worst)

____ Having a friend or brother/sister do it with them

____ Go to a fitness center or site that other kids go to

____ Get better at their sport, dance, martial art, or other activity

____ Trying new activities

____ Receive rewards

____ Other:

28. Rank order the list of rewards your child may prefer: 1 (best)–7 (worst)

____ Free game apps for their phone or computer

____ Favorite food treats like

____ Free tickets to a movie, play, or concert

____ Free entry into an amusement park

____ Free months at the gym, dance program, martial arts program or other

____ Free gear for participating in activity of choice

____ Other rewards: ___________________________________________

29. Would you or a caretaker be willing and able to drive your child to a community-based program?

(Select one)

____ Yes (1)

____ No (2)

30. Would it be more feasible for your child to participate in a community-based program if we provided transportation?

(Select one)

____ Yes (1)

____ No (2)

31. Do you know what the current recommendation is for your child to exercise to maximize their bone and muscle health?

____ Yes (1)

____ No (2)

Display: *Do you know what the current recommendation is for your child to exercise to maximize their bone and muscle health = Yes or No*

32. If you answered yes to the question above, what frequency are you aware of?

________________________________________________________________

________________________________________________________________

33. How many days per week could your child attend a fitness program, outside your home, considering your schedule, your child’s other activities, rides, etc. Rank 1 (most likely)–4 (least likely)

______ 1x/week

______ 2x/week

______ 3x/week

______ More

34. What would be your preferred frequency if there were few obstacles such as transportation or other obligations? Rank 1 (best)–4 (worst)

______ 1x/week (1)

______ 2x/week (2)

______ 3/week (3)

______ More (4)

35. How long could your child realistically participate given current constraints (rank order)?

______ 1 month

______ 2 months

______ 3 months

______ More

36. What is the biggest challenge for your child to participate in a regular fitness program?

________________________________________________________________

________________________________________________________________

________________________________________________________________

37. Other Comments:

________________________________________________________________

________________________________________________________________

________________________________________________________________

________________________________________________________________

**End of Block: Part 2: Caretaker**
Kimbel, J. D.; Duff, S. V.; Friel, K. M.; Grant-Beuttler M.; Sukal Moulton, T.; Moreau, N. Incentives to Participate in Fitness Programming: Insights From Youth and Parents. *Qualtrics*. **2020**.
